# Conceptional design for a universal HVDC-HVAC vacuum-interrupter-based circuit breaker

**DOI:** 10.1038/s41598-024-69840-3

**Published:** 2024-08-27

**Authors:** Fady Wadie, Tamer Eliyan

**Affiliations:** 1 Mechatronics and Robotics Engineering Department, Faculty of Engineering, Egyptian Russian University, Badr City, Egypt; 2 Faculty of Engineering at Shoubra, Benha University, Cairo, 11629 Egypt; 3 Department of Electrical Power and Machines Engineering, The Higher Institute of Engineering at El-Shorouk City, Alshorouk Academy, Cairo, 11837 Egypt

**Keywords:** Electrical and electronic engineering, Energy infrastructure

## Abstract

Modern power systems high voltage transmission systems either HVDC or HVAC has mandated the presence of two types of circuit breakers (CB), HVAC-CB and HVDC-CB. That required two different production lines, higher costs and more complicated manufacturing process. A solution is proposed in this paper which is a concept design for a universal HVCB (UHVCB) that is applicable to both HVDC and HVAC system. Such a concept would allow a faster, easier production and more economical unit cost for CBs that would benefit the entire industry from manufacturers to utilities. The design of HVDC-CB was used as the foundation the proposed UHVCB such that L–C branches are used. The UHVCB was tested in both HVAC and HVDC transmission systems. The results showed the reliable performance of UHVCB in both systems. The recorded transient recovery voltage (TRV) was reduced from 750 to 430 kV when UHVCB was used instead of conventional HVCB. The testing included both technical and economic aspects. The performance of the UHVCB was tested by varying the parameters for L–C shunt branches in both systems. That included varying the value of L in range 0.3–1 mH and 10–30 µF for C. The most important conclusion from this paper that a UHVCB that is applicable in HVDC and HVAC systems is achievable and this paper is only an initial step in achieving this goal.

## Introduction

High voltage power transmission has thrived during the recent decades as a part of the global co-operation to solve the increasing energy problem. The global solution was transformation to green energy based power generation. The intended use of high voltage transmission was the transmission of bulk power across vast distances across wide geographical areas for some countries and internationally between different countries. That types of transmission still faces different challenges including power losses over large distances, geographical obstacles and unsynchronized power systems^1–3^. High voltage direct current (HVC) transmission has shown great potential in that field during the last decades. This is attributed to its ability to minimalize power losses over vat distance transmission in addition to its inherited advantage of connecting unsynchronized power systems^4–7^. The protection of HVDC systems is founded on its main tool for switching and protection actions; the circuit breaker^8,9^.

The circuit breakers (CBs) for HVDC system has been studied tremendously in literature for the un-paralleled challenge it faces unlike its AC counterparts, which is the lack of a natural zero-crossing for currents^10–12^. To overcome this problem, HVDC CBs use an artificial zero-current-crossing technology via an L–C shunt element. That element injects an oscillating current from the interactive interchange of energy between its L and C components^13–16^. The oscillating current superposition the CB current allowing the current to cross the zero point giving a chance to the CB to interrupt the switching arc. Therefore, the design of HVDC included a mechanical interrupting element as the main switching element and L–C shunt element. Additional shunt elements included Resistive branch to provide damping effect that limits the rate of rise of transient recovery voltage and metal oxide variastor to absorb the injected fault energy^15–18^. The mechanical interrupter element employs an arc-quenching medium either gaseous as SF6 CBs or vacuum in interrupters (VIs). The main focus of this paper will be on VIs.

The other side of high voltage transmission is HVAC transmission which might be an unavoidable way of connection in countries with vast distances between its cities or having geographical limitations^19–21^. These HVAC systems require a HVAC CBs equipped with high withstanding dielectric strength. That challenge is considered a common part for application of VIs as CBs in HVAC or HVDC. For such reason, one of the main factors considered during CB selection is its rate of rise of dielectric strength (RRDS). To elevate the RRDS for VI-based CB so that it could fit in HVAC or HVDC environments, a multi-chamber or multi-break VIs are used^22,23^. The ability of VI’s RRDS to withstand the transient recovery voltage (TRV) appearing across the CB after initial arc interruption is a very crucial topic. If the TRV exceeds the RRDS of VI, the arc is re-ignited. The VI would still be able to extinguish the arc but if the RRDS is still inadequate to withstand the TRV, the arc would re-ignite again in a phenomenon known as multiple arc re-ignition^24–29^. Even though multiple arc re-ignition for VIs has been widely studied AC systems^26–29^, but their consideration in HVDC systems within VIs was limited without being considered in researches^30–36^ with rare consideration as in ^37,38^. The HVDC-CB was investigated from many researchers reaching to interruption parameters of fault current of 8 kA on a 400 kVA^39^.That was later extended to included modularized series VIs in^40^. That continuous advancement in HVDC CB technology was formulated in^41^. A summary based on chronological order for VI-based switching literature is presented in Table 1. The summary clearly visualizes the lack of combined study for HVDC and HVAC system with consideration of multiple arc-reignitions in both systems. Hence, the main problem of the paper could be formulated as studying the multiple arc-reignitions in both HVDC and HVAC environment and providing a concept for a design of a universal high voltage circuit breaker UHVCB based on VI that could be used in HVDC and HVAC systems. Based on the previous, the main contributions of this paper could be highlighted as follows:

1. Investigating the phenomenon of multiple arc-reignitions for VI-based HVDC and HVAC-CBs.
2. Providing a concept for a universal design for HVCB that could be applicable for both HVDC and HVAC systems.
3. Studying the ability of the provided concept in reducing multiple arc-reignitions in HVDC and HVAC systems.
4. Present a study for the impact of the design elements (L, R and C) of the UHVCB in reducing the arc-reignitions.
5. The main contribution of this paper and for UHVCB will be its impact in the manufacturing process of the CB and final pricing of the CB. As reaching a design for UHVCB that could be used for HVDC or HVAC systems allows manufacturers to have one large scale production line for UHVCB instead of two production lines, one for HVDC-CB and the other for HVAC-CB. That would result in a faster rate of production, increased efficiency in various parts of the manufacturing process including; faster assembly process, lower time losses in logistics, easier management of the supply chain and generally a more economical production. The whole process would benefit the manufacturers and that would allow them to provide UHVCB with a lower unit prices than regular forms of either HVDC or HVAC CBs.

**Table 1:** Chronological Summary for VI-base switching in different environments.

Ref	Year	Environment of the study			Arc-reignition considered
		MVAC	HVAC	HVDC	
26	2023	✓	–	–	✓
27	2023	✓	–	–	✓
29	2022	✓	–	–	✓
31	2022	–	–	✓	–
25	2021	✓	–	–	✓
20	2020	–	✓	✓	–
24	2020	✓	–	–	✓
38	2020	–	–	✓	✓
22	2019	–	–	✓	–
23	2019	–	✓	–	–
37	2018	–	–	✓	✓
19	2016	–	✓	✓	–
21	2008	–	✓	–	–

The limitations of this work will include technical and economical limitations. Technical limitations will include the ability to combine the requirements of both systems into a single type of CB. Economical limitations will be reaching a more reduction in cost without compromising the technical targets of the CBs. The advantages of using the proposed universal HVCB over regular AC or DC CBs are the increased production speed due to focusing in a single form of CB, increasing production rate and subsequent reduction in production costs lower and that would eventually lead to a lower unit cost of CBs. Hence, this benefits the manufacturers, the utilities and the consumers. The disadvantages will be the increased L–C branches which could be handled by re-modifying some the accepts of VI while maintaining the technical performance of CB intact. That way both technical and economic aspects could be met. The remainder of this paper is presented as follows. The proposed concept of UHVCB is proposed in “Concept design for universal high voltage circuit breaker” section. Section “Modeling of vacuum interrupter” navigates through the modelling process of the VI-based UHVCB using ATP/EMTP software. Section “Systems under study” presents two testing systems; an HVDC and HVAC transmission lines which were used in testing the UHVCB. Results from the simulation process are presented in “Simulation results” section. Finally, conclusions are drawn in “Conclusions” section.

## Concept design for universal high voltage circuit breaker

**Figure 1:**
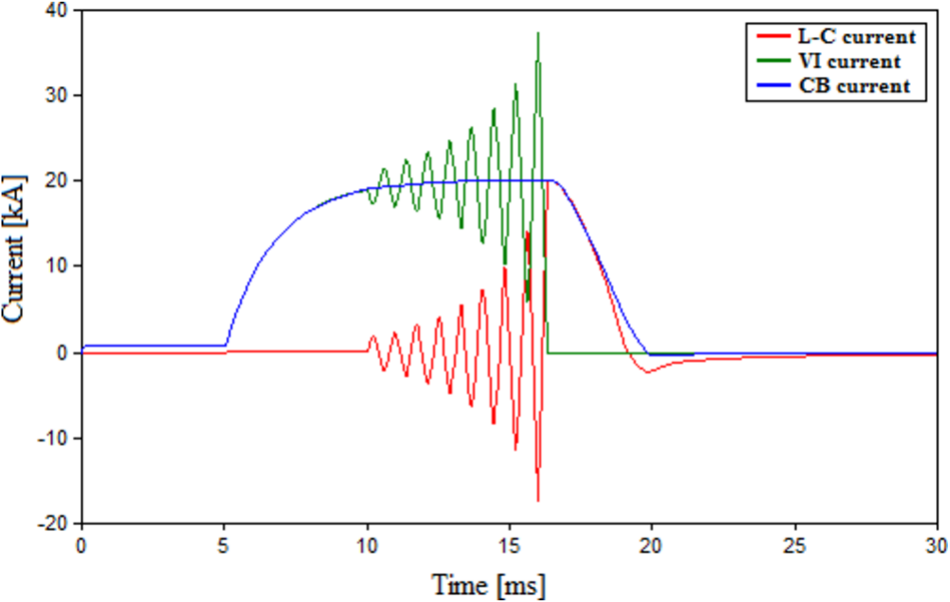
Currents flowing VI, L–C branch and CB.

The main component for high voltage circuit breaker is the interrupter that is considered using vacuum interrupters (VIs) in this study. VIs depend on the their RRDS to build up a suitable dielectric strength to withstand the TRV. The withstanding of the VIs presented lately could reach up to 200 kV^37^. For the testing systems in this study of 500 kV, multi-chambers VI-based HVCB are used which consist of eight series VI chambers which are assumed to share the system voltage equally^37^. Therefore, the design for HVCB in HVAC system would include multiple chamber suitable enough to withstand the system voltage. For HVDC systems, the lack of a natural current zero crossing is compensated by using a shunt LC element that injects an oscillating current and forces artificial current zero to interrupt the arc. A resistive branch and MOV are also used to control the rate of rise of TRV and absorb excessive arc energy respectively. The role of L–C in injecting a zero-current crossing could be more explained as follows. When current through the L–C branch, an oscillatory current is injected and gets in superposition with the VI-current. That causes the total breaker to have an oscillating effect till it reaches a zero-crossing termed as artificial zero crossing. To further clarify on the action of L–C to generate artificial zero-current crossing, the currents flowing through a typical VI, the shunt L–C branch and their total CB current are shown in Fig. 1. When a fault occurs at 5 ms in the figure both VI and CB current rise because of the fault. The CB sends a trip signal and tend to interrupt the fault. That signal connects the L–C circuit at 10 ms and at that instant the oscillatory effect of the L–C current is noticed in Fig. 1. Such effect gets in superposition with the interrupter current till they reach zero-crossing at approximately 16 ms. That is followed by a decay in total CB current till the arc is interrupted. Hence, it is clear that the oscillatory effect of L–C circuit allowed the CB to get an artificial zero-crossing and interrupt the arc.

Hence the main difference between HVDC and HVAC CB is the additional LC, R and MOV associate with VI in HVDC CB. To formulate UHVCB that combines both designs into a single design for both systems; two possibilities are available:

1. Eliminate the additional branches in HVDC-CB so it could usable in HVAC systems, or
2. Add LC and R branches to HVAC CB so it is usable in HVDC systems.

The first possibility is inapplicable as eliminating the additional branches would prevent the HVDC-CB from reaching a current zero. Hence, the second possibility is considered to be more achievable. Therefore, the first step for reaching a design for UHVCB is defining the main frame for the CB which is:

1. Multi-chamber VI
2. LC branch
3. Resistive branch

**Figure 2:**
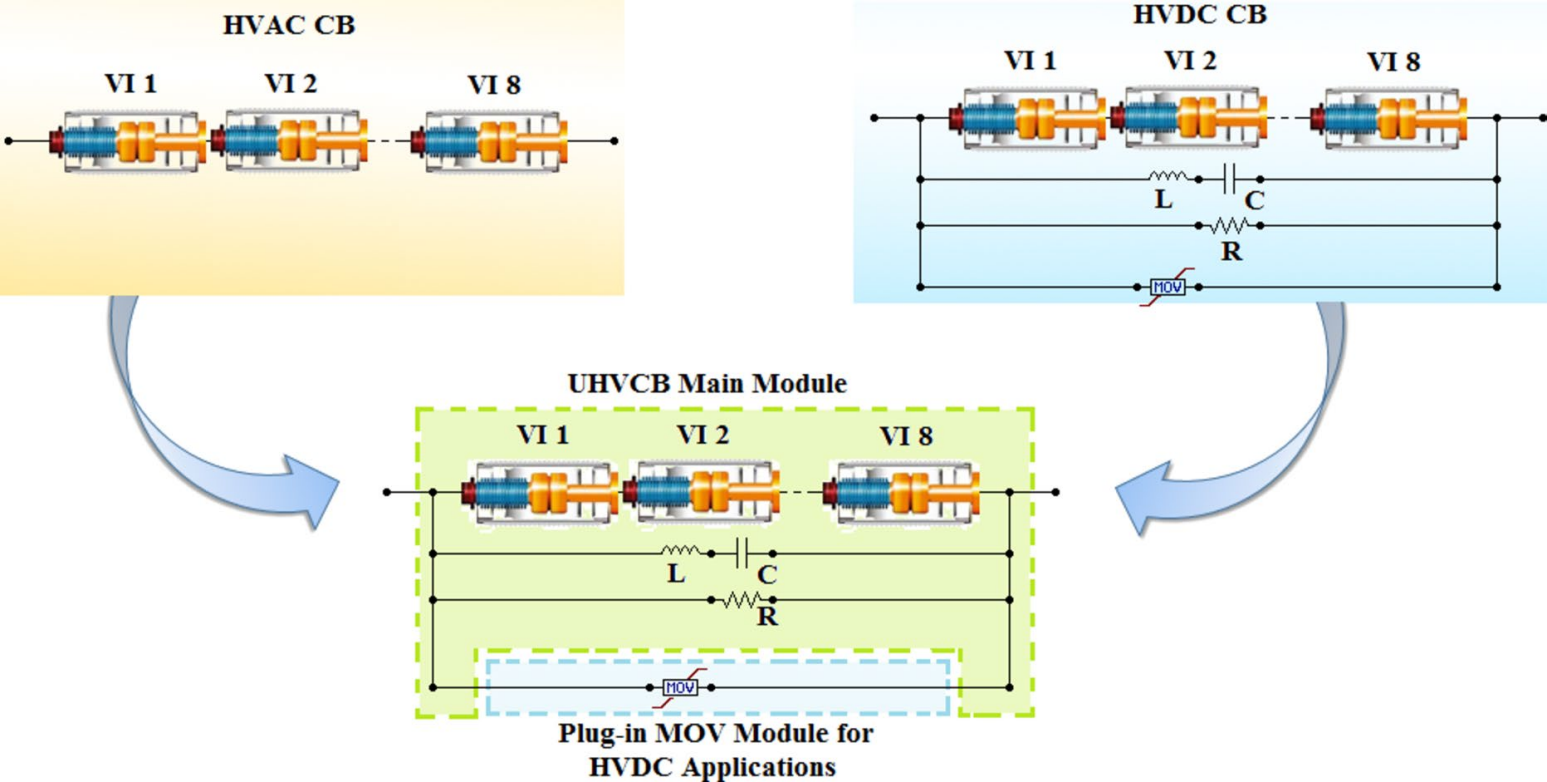
Schematic of HVAC-CB, HVDC-CB and the proposed UHVCB.

All of them to form a UHVCB that could be used in HVDC and HVAC systems as shown in Fig. 2. The only part to be added will the MOV. The standard module will be made with an empty compartment for the MOV that could be added if the UHVCB is to be used in HVDC systems, otherwise that compartment will be left empty. The performance for the proposed UHVCB in HVDC systems is expected to be that of a regular HVDC-CB as this design is based upon the standard design of HVDC-CB. The evaluation of performance of the proposed UHVCB design in HVAC could be made from a technical and economical point of view as follows:

- From the technical point of view, the added branches will enhance the performance in HVAC system as LC branch will help reach faster a zero crossing in AC currents allowing a faster interruption.
- From economical point of view, the added branches will represent an added cost. That cost could be compensated by using of a VI of lower RRDS.

For example, in a certain HVAC system, the RRDS of the HVCB used is 50 V/μs to reach a satisfactory result during switching. The addition of the LC and R branches as a part of UHVCB design will enhance the performance and increase the unit cost for the CB. To avoid increasing the unit cost, the VI could be replaced with a VI of 10 V/μs which would be of lower unit cost than original 50 V/μs VI. The performance would not get affected by that replacement as 10 V/μs is associated with LC and R branches that would relatively reach similar performance to the standalone 50 V/μs VI. That comparison is tested and presented in the simulation section. In summary, it could be said that the design of UHVCB uses conventional HVDC design, and for HVAC, it compensates the increased branches by using VIs if lower RRDS to maintain the same unit cost.

This design is tested in simulation section to define the following:

1. Ensure that using lower RRDS with shunt branches reaches the same performance as a standalone VI with higher RRDS.
2. Ensure the acceptable performance in both HVDC and HVAC systems.
3. Define the appropriate parameters of shunt branches.

## Modeling of vacuum interrupter

The modeling process of VI is inheritably attached to the modeling of multiple arc re-ignitions generated during the switching of VI. That is achievable through a controlled switch which could be closed to represent the state of the arc presence or opened to represent the arc extinguishing phase. To control the switch, a basic program is built using ‘MODELS’ component available upon ATP software package. The built program follows the sequence of events following the switching process and defines the state of VI either arc is isolated or re-ignited based on mathematical conditions. The sequence following the receiving of the trip signal to the VI and the mathematical modeling are presented as follows^42^:

- At the initial phase of mechanical interruption, the arc is present during that initial phase and the switch model is closed to model that phase.
- When the current across the VI reaches the current chopping value, the VI interrupts the arc and the switch model is opened. The average chopping current is shown in (1) ^42^.
  1 $$ \overline{{{\text{I}}_{{{\text{ch}}}} }} = \left( {\upomega {\hat{\text{i}}} \upalpha  \upbeta } \right)^{{\text{q}}} $$
  where $\upomega = 2\uppi \left( {50\;{\text{Hz}}} \right)$, ${\hat{\text{i}}}$: amplitude of the 50 Hz current, ${\upalpha } = 6.2{ } \times 10^{ - 16} \;{\text{s}}$, ${\upbeta } = 14.3\;\;{\text{q }} = \left( {1 - {\upbeta }} \right)^{ - 1}$.
- A TRV arises across VI following the arc-interruption. If that TRV surpasses the RRDS of the VI, the arc is re-ignited and the switch model is closed to represent the re-ignited arc. The RRDS is computed from (2)^42^.
  2 $$ {\text{U}} = {\text{A}}\left( {{\text{t}} - {\text{t}}_{{{\text{open}}}} } \right) + {\text{ B}} $$
  where U: the withstand voltage, ${\text{t}}_{{{\text{open}}}}$:the moment of contact separation, A: Manufacturer’s parameter for rate of rise of dielectric strength taken which could range from 10 to 50 V/μs, B: Breaker’s TRV just before current zero and in this study B was considered as zero.
- The VI could re-interrupt the arc if its high frequency current quenching capability HFQC exceeds the rate of change for the current at zero crossing. The model switch is reopened. The HFQC is given in (3)
  3 $$ {\text{HFQC }} = {\text{E}}\left( {{\text{t}} - {\text{t}}_{{{\text{open}}}} } \right) + {\text{D}} $$
  where E: Rate of rise of the HFQC of the VCB and E is considered as 600 A/μs^2^, D: is HFQC of the VCB just before contact separation and D is considered zero in this study^42^.

The validation for the model was conducted via the testing circuit provided in^43^ and generated model showed to provide the same results as those presented in^43^. The second part of the VI model is the resistive part which represents the inverse of the arc conductance state^44,45^. In the closed state, the resistive value is low in μΩ range while in open state, a high value is reached of 1 TΩ^44,45^.

## Systems under study

### HVAC transmission system

The testing HVAC transmission system is based on El-Kuorimate – Cairo 500-kV, 124 km transmission line. The systems data are provided in Table 2^46^. ATP’s LCC JMarti model were used to simulate the overhead transmission line that utilizes Frequency dependent model with constant transformation matrix.

**Table 2:** HVAC Transmission line data.

Positive and negative sequence impedance per phase in ohm	3.307 + j14.053
Zero sequence impedance per phase in ohm	10.75 + j45.67
No. of sub-conductors per phase	3
No. of ground wires	2
Diameter of sub-conductor in mm	30.6
Vertical height of conductor at mid-span in meter	9
Vertical height of ground wire at mid-span in meter	21
Diameter of ground wire in mm	11.02
DC resistance of conductor in ohm per km	0.0133
DC resistance of ground wire in ohm per km	0.35
Ground tower rod length in meter	1.5
Ground tower rod radius in cm	1.25

### HVDC transmission system

**Figure 3:**
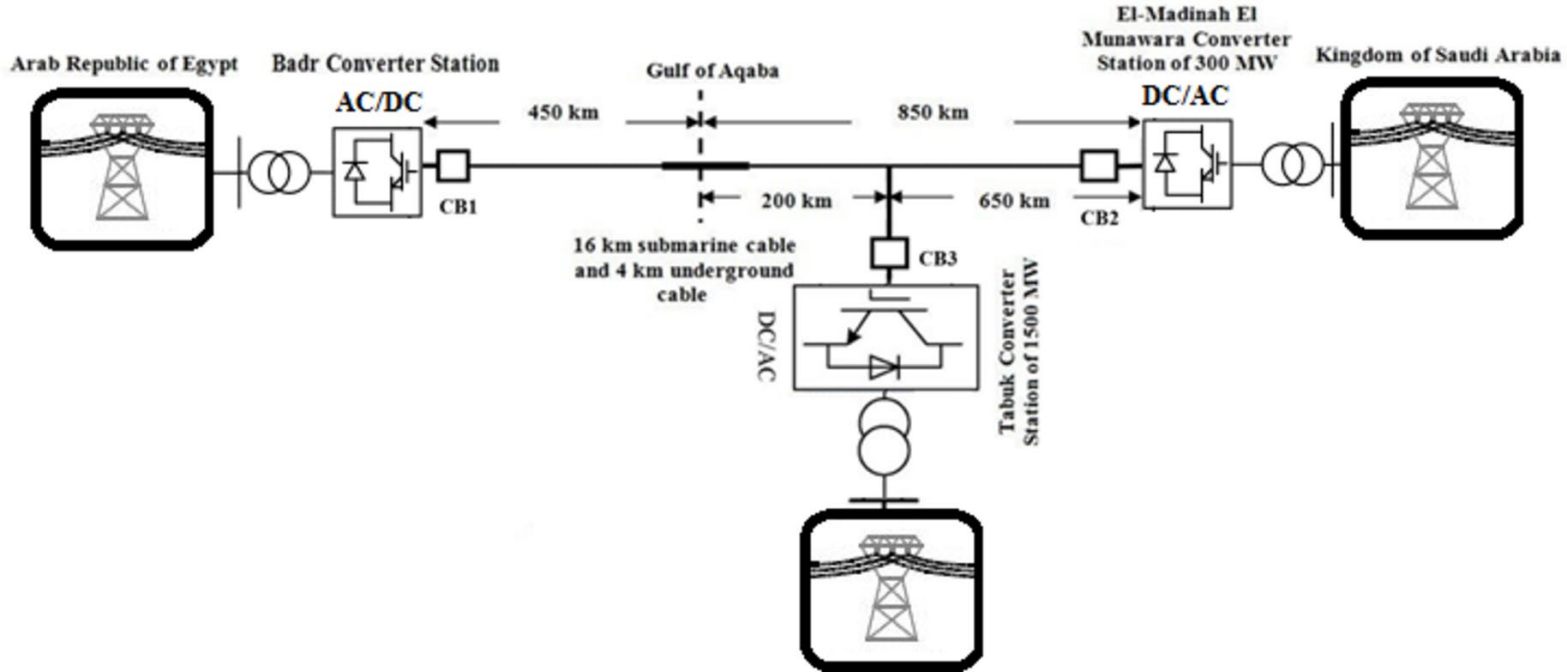
The HVDC transmission representation of the studied practical system between Egypt and Saudi Arabia.

The HVDC testing system is selected as a part of a real HVDC interconnection project between Egypt and the Kingdom of Saudi Arabia that is currently under construction. The project includes consists two 500 kV AC/DC substations, a linking station, and a 1300 km transmission as shown in Fig. 3. A portion of the project was selected to be the focus of study. That portion is a 450 km line located in Egypt and connects Badr substation and Elnabaq switching station presented. That part operates at 500 kV HVDC^18^. A 500 kV source is used to model the selected system as a power supply providing it to 650 Ω load^18^. The selected portion of the overhead HVC transmission line is modeled as a 24.5 Ω resistor and 45 mH inductor connecting the source and the load^18^.

## Simulation results

The simulation process utilizes the usage of UHVCB as described in previous sections and testing it into two main systems; HVAC and HVDC testing systems that were described in the testing systems section. For each system, the UHVCB will be used in switching the circuit and the resulting TRV or arc re-ignitions will be recorded to evaluate the performance of the CB. In addition, in the HVAC system an additional point will be added. That point would to compare the performance of the conventional HVAC-CB with respect to the performance of the proposed UHVCB. Finally, the suitable L and C parameters are defined for the UHVCB based on its performance in both systems.

### HVAC simulation results

#### Case 1: using 50 V/µs-VI-based HVAC-CB

**Figure 4:**
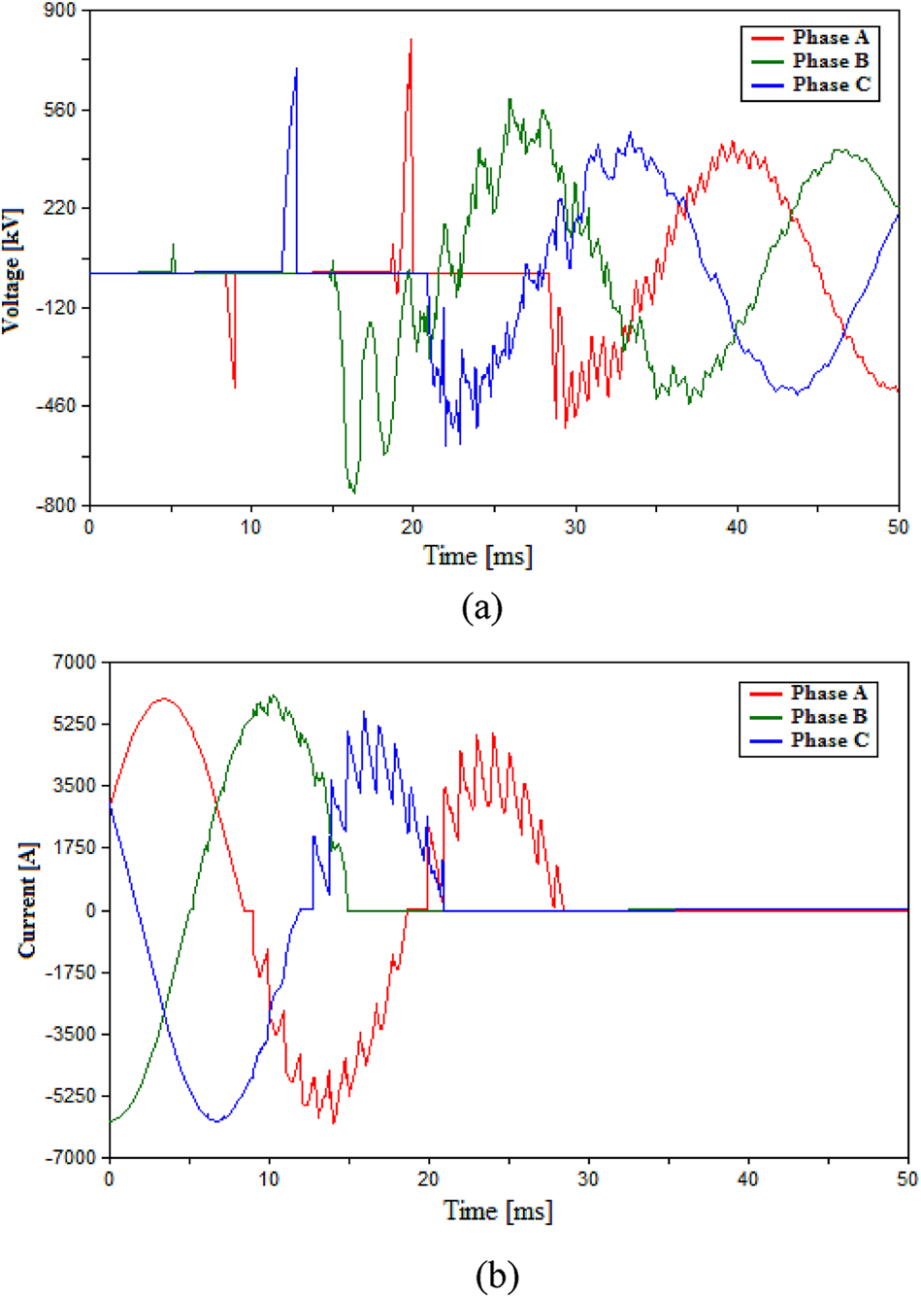
Switching in HVAC transmission system using conventional HVAC-CB with RRDS = 10 V/µs (**a**) Voltage across VIs (**b**) Current flowing through CB.

**Figure 5:**
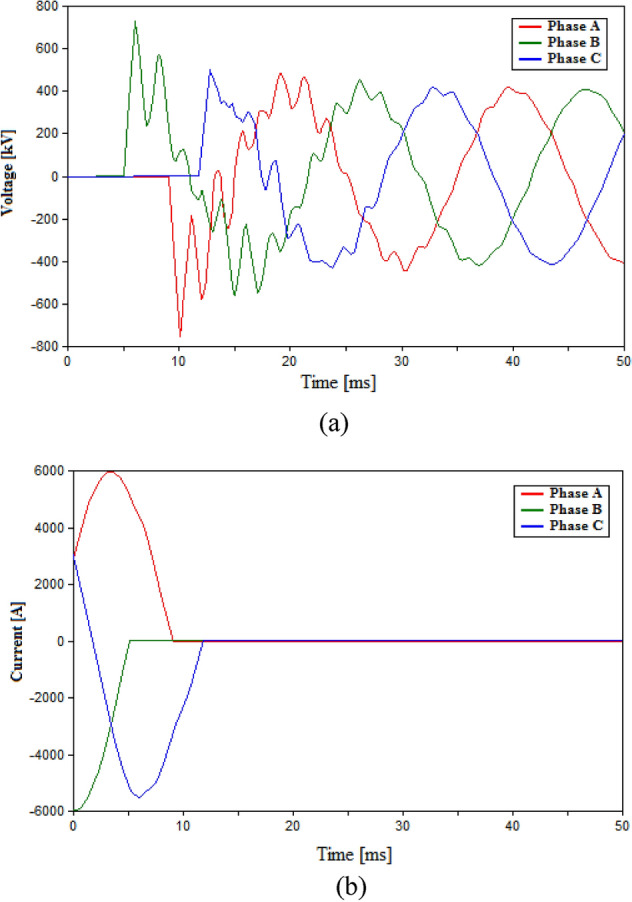
Switching in HVAC transmission system using conventional HVAC-CB with RRDS = 50 V/µs (**a**) Voltage across VIs (**b**) Current flowing through CB.

The first case in the testing process within the HVAC system will be using a conventional HVAC-CB consisting of eight VI chambers. Since the conventional HVAC-CB does not include any additional elements as L and C, so only the RRDS will be varied during this case. Two RRDS values are tested which are 10 V/µs and 50 V/µs for the 500 kV HVAC system and their results including the voltage across the VIs and the current flowing through the CB are shown in Figs. 4 and 5 respectively. Numerical recordings for the results are presented in Table 3. The results of Fig. 4 for 10 V/µs case, show the occurrence of multiple arc-reignitions and TRV reaching 797.8 kV. While Fig. 5 show the successful opening of the CB with no arc-reignitions recorded for the 50 V/µs case. The maximum recorded TRV for the layer case was 752 kV as presented in Table 3. Hence, for conventional HVAC-CB the reasonable choice for operation will be using VIs with RRDS of 50 V/µs. Therefore, the results from conventional 50 V/µs-based HVAC-CB will be used in comparison with those from the UHVCB to evaluate to the performance of the UHVCB.

**Table 3:** Simulation results in HVAC transmission system.

Parameters of CB			Maximum recorded TRV (kV)	Arc re-ignition
RRDS (V/µs)	L (mH)	C (µF)		
50	–	–	752.7	No
10	–	–	797.8	Yes
10	0.3	20	431.02	No
10	1	20	431.33	No
10	10	20	434.93	No
10	0.3	5	395.3	Yes and Phase B failed to open
10	0.3	40	433.2	No

#### Case 2: using 10 V/µs-VI-based UHVCB

**Figure 6:**
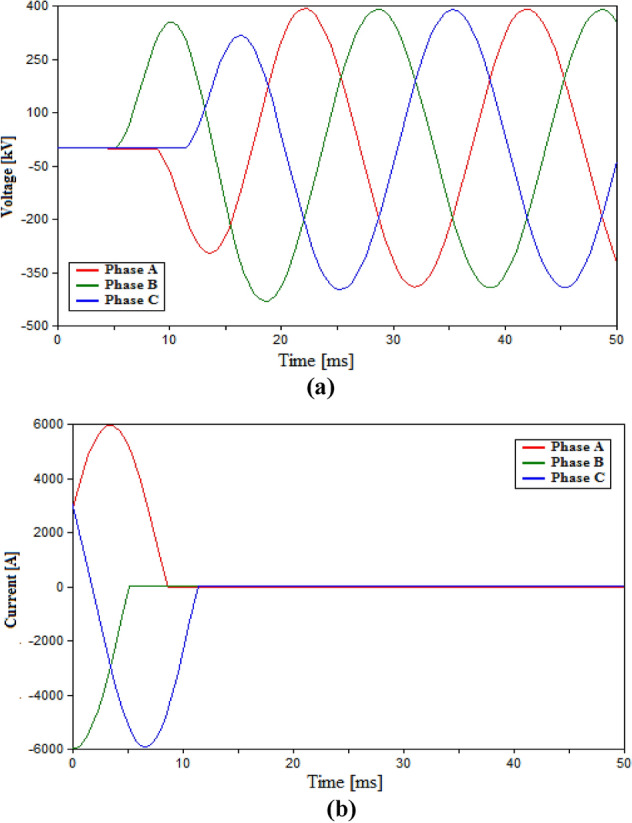
Switching in HVAC transmission system using UHVCB with RRDS = 10 V/µs, L = 0.3 mH, C = 20 µF, R = 150 Ω (**a**) Voltage across VIs (**b**) Current flowing through CB.

The second case in the testing process within the HVAC system will evaluate the performance of the proposed UHVCB in HVAC transmission systems. The UHVCB will have the structure earlier mentioned; which includes the eight VI chambers associated with LC and resistive branches. As earlier discussed, the RRDS for the UHVCB will be lower than conventional HVAC-CB in an attempt to compensate for the additional cost for the shunt branches. Hence, the main aim of this testing case that the UHVCB with lower RRDS still provide reasonable switching performance as that provided by conventional HVAC-CB with higher RRDS. The RRDS for the UHVCB in this case is selected to be 10 V/µs such that it is lower than 50 V/µs conventional HVAC-CB tested in previous case. The added L–C branch will have 0.3 mH and 20 µF respectively. These values will be varied in later testing process to examine their effect on the results. The damping resistive branch is set to 150 Ω. The results for the voltage and current of CB are shown in Fig. 6 and numerically in Table 3. The results from figure show that the UHVCB have opened successfully with no re-ignitions and TRV reaching only 431 kV as given n Table 3. These results could be compared to those for conventional HVAC-CB of 50 V/µs shown in Fig. 5. It is evident from this comparison, that UHVCB with RRDS of 10 V/µs provide a similar or even better performance than those from conventional HVAC-CB with RRDS of 50 V/µs. As the UHVCB successfully opened with no re-ignitions and even allowed a lower TRV than those from conventional HVAC-CB with higher RRDS. Therefore, it is clear the UHVCB is capable of providing a suitable performance even with lower RRDS than conventional CBs that the allowing the total per-unit cost to remain reasonable and allowing the benefits from wide scale production for UHVCB to be more effective in terms of pricing.

**Figure 7:**
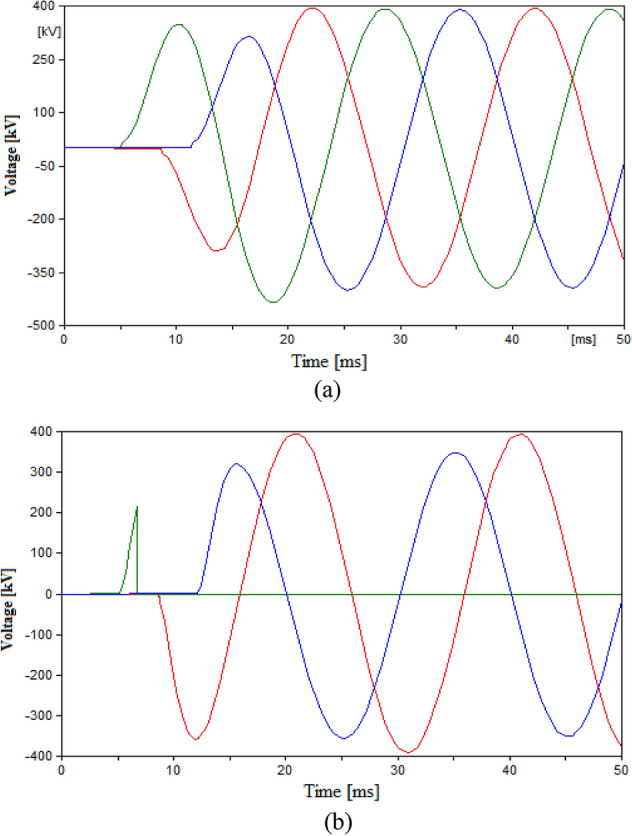
Switching voltage in HVAC transmission system across the UHVCB with RRDS = 10 V/µs and LC parameters (**a**) L = 10 mH, C = 20 µF (**b**) L = 0.3 mH, C = 5 µF.

At this point, two parts of the aims of the simulation are already fulfilled which are testing the performance of UHVCB in HVAC systems and ensuring that lower RRDS will provide suitable performance as that from conventional CB. The remaining aims within this case is to define the suitable L–C parameters for the UHVCB. The values of L and C will be changed in different cases and the resulting TRV or re-ignitions will be recorded. The value of L was to 1 mH and 10 mH while keeping other parameters unchanged as recorded in Table 3. The recorded TRVs were slightly increased to 431.33 kV and 434.93 kV respectively with no re-ignitions recorded. Hence, the 0.3 mH will be considered a satisfactory value for L. When the value of C was changed to 5 µF, the results showed multiple re-ignitions and failure to open in phase B. The increase in C value to 40 µF showed a successful opening with no re-ignitions but the TRV reached 433.2 kV. Hence, the 20 µF is considered a suitable value for C. The results for the case of L = 10 mH, and case of 5 µF capacitance are shown in Fig. 7.

### HVDC simulation results

**Figure 8:**
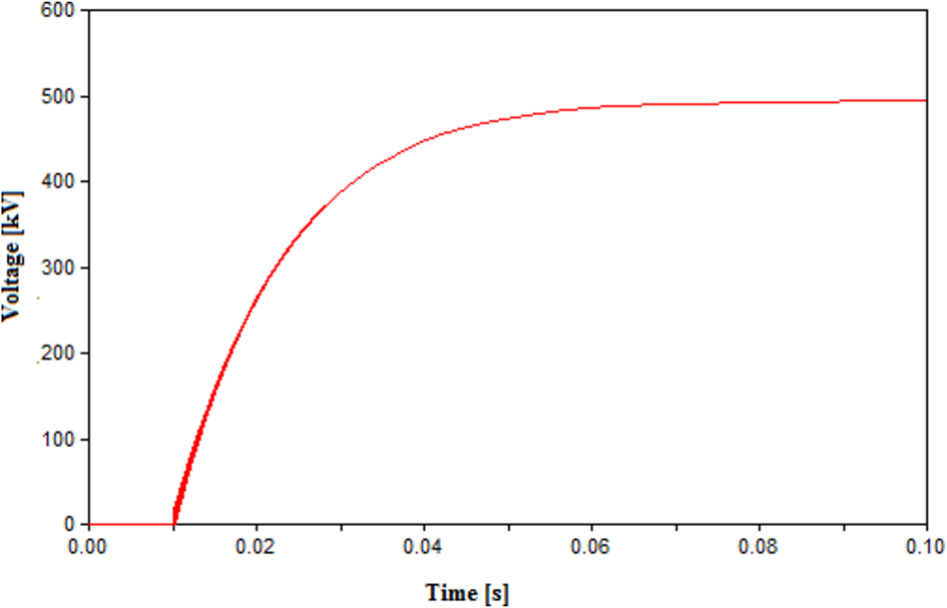
Switching voltage in HVDC transmission system across the UHVCB with RRDS = 10 V/µs and LC parameters L = 0.3 mH, C = 20 µF.

The testing sequence for HVDC systems differs from that in HVAC system in not requiring to the compare the performance of UHVCB with conventional HVDC-CB. That is because the design for UHVCB is based on the design of HVDC-CB. The only requirement for UHVCB to be used in HVDC system is to insert the MOV in its plug-in compartment within UHVCB as shown in Fig. 2. The UHVCB equipped with MOV is tested in this subsection within the HVDC testing system earlier detailed. The main aim of this part will be to evaluate the performance of UHVCB in HVDC systems and define the suitable L–C parameters for its application during switching. The results for the voltage across the UHVCB with L of 0.3 mH, C = 20 µF and R = 150 Ω. The result shows successful opening of the CB with no reignitions recorded as see in Fig. 8.

**Figure 9:**
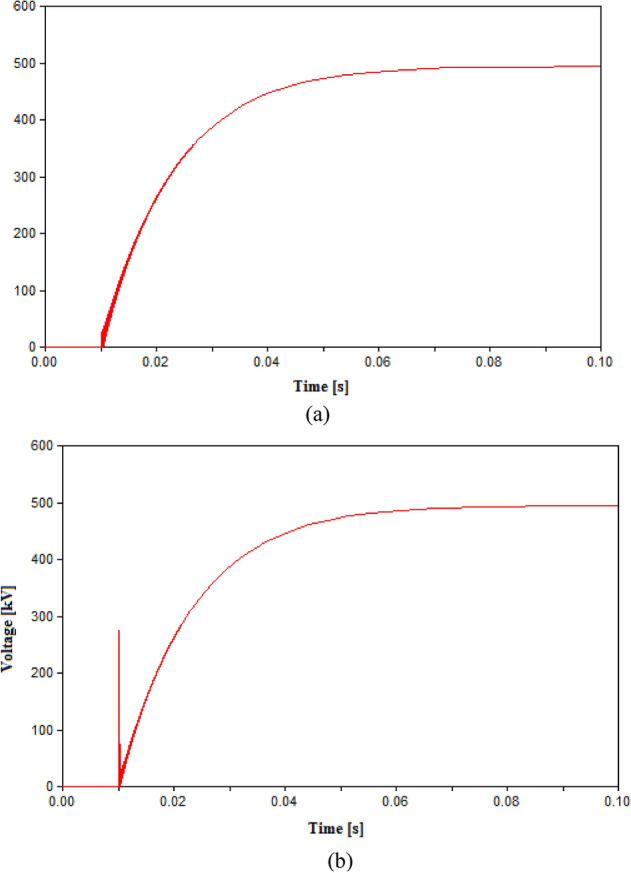
Switching voltage in HVDC transmission system across the UHVCB with RRDS = 10 V/µs and LC parameters (**a**) L = 1 mH, C = 20 µF (**b**) L = 10 mH, C = 20 µF.

**Figure 10:**
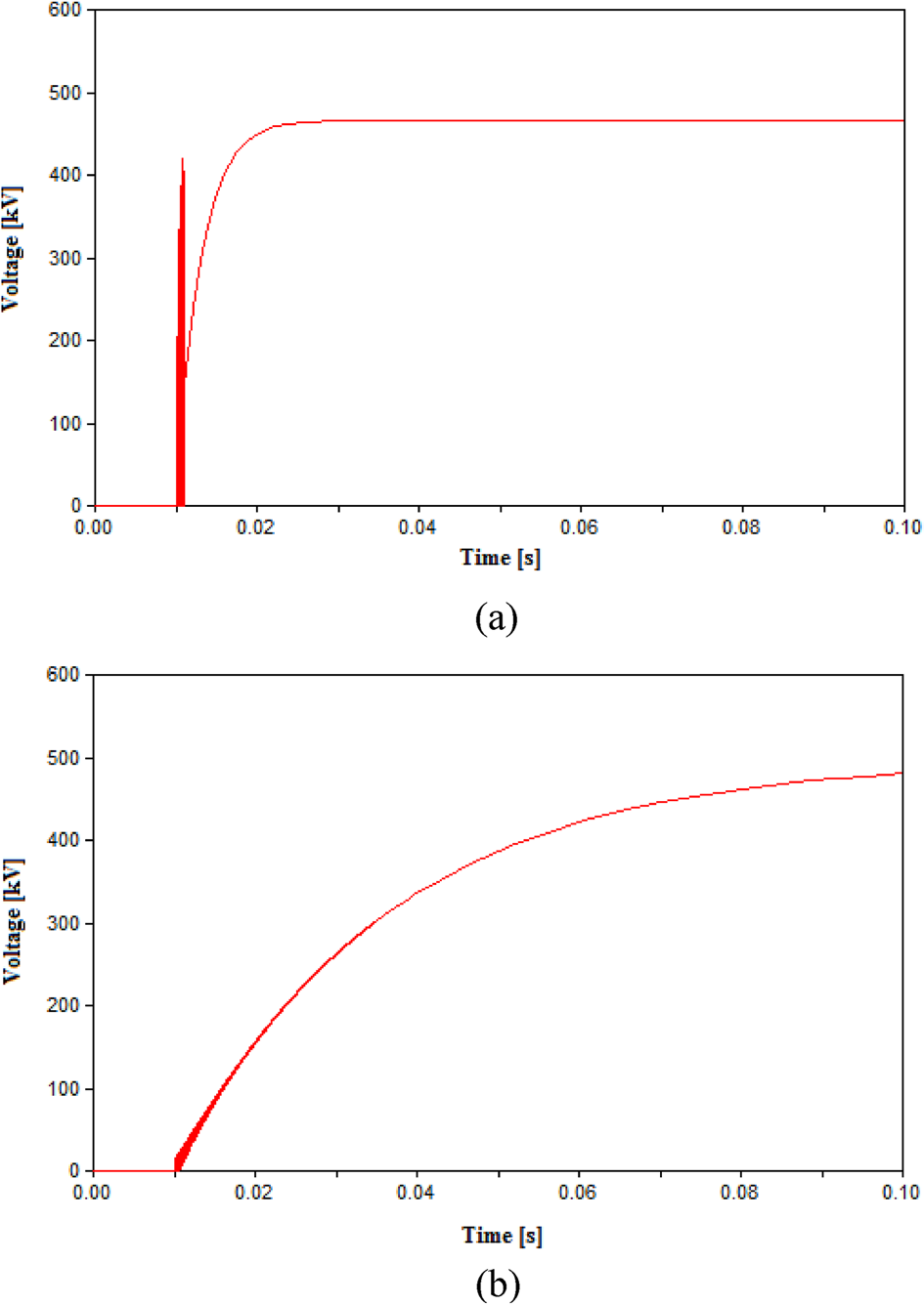
Switching voltage in HVDC transmission system across the UHVCB with RRDS = 10 V/µs and LC parameters (**a**) L = 0.3 mH, C = 5 µF (**b**) L = 0.3 mH, C = 40 µF.

The impact of L and C was studied by changing L to 1 and 10 mH, and C to 5 µF and 40 µF as shown in Figs. 8 and 9 respectively. The change in L to 1 mH had little impact on the final result, however for 10 mH the sudden voltage spike appearing at the instant of tripping at 10 ms as shown in Fig. 9. The spike recorded at 10 ms is due the sudden change in current leading to a high induced voltage as an action of the inductance L. This spike is noticed at high inductance value as the induced voltage is directly proportional to the L and rate change of current. Therefore, it is reasonable to used inductance L in range from 0.3 to 1 mH. The impact of changing the capacitance showed that reducing C to 5 µF lead to multiple re-ignitions at the instant of tripping as shown in Fig. 10a. While increasing the capacitance avoided these re-ignitions but led to a larger time constant and hence, reaching the steady state value took much larger time than other cases as shown in Fig. 10b. Therefore, it is concluded that the 20 µF is a reasonable value for the capacitance. This result is subject to change based on the parameters of the HVDC system, hence as guiding step for other systems a range for possible values of C could be tested of 10–30 µF.

## Conclusions

The main motivation behind this work is the need for providing a reasonable pricing for HVCB. The proposed criteria to achieve this aim was providing a concept design for a universal HVCB or UHVCB that is applicable to both HVDC and HVAC systems. That would allow manufacturers to have a single production line for UHVCB rather than two production lines one for HVDC-CB and HVAC-CB. Achieving this target would lead to lower production cost, faster assembly and lower per-unit price that would benefit both manufacturers and utilities. The proposed design for UHVCB was based on the design of HVDC-CB where L–C shunt branch are included in the design. The proposed UHVCB was test into two real systems of HVDC and HVAC transmission using ATP/EMTP software package. The results of the simulation lead to following conclusions:

1. The performance of VI-based-UHVCB of RRDS = 10 V/µs in HVAC systems was better than conventional HVAC-CB with higher RRDS of 50 V/µs. This was based on TRV recorded which reached a range of 430 kV and 750 kV for UHVCB and HVAC-CB respectively.
2. The performance of UHVCB of lower RRDS of 10 V/µs in HVAC systems is acceptable and the cost of additional shunt branches is compensated by using lower RRDS while keeping a suitable switching performance.
3. The performance of UHVCB in HVDC system was acceptable with no-reignitions recorded based on L–C parameters.
4. The suitable parameters range for L and C from both HVDC and HVAC could be in range 0.3 to 1 mH and 10–30 µF for L and C respectively.
5. The defined L–C parameters are based on the testing systems parameters and could be subject to variation based on the chosen systems.
6. The most important conclusion that based on all previous conclusion is that reaching a universal type for HVCB that is applicable in HVDC and HVAC systems is achievable and this paper is only an initial step in achieving this goal.

## Data Availability

Data is provided within the manuscript.
